# Supplementary material for: Are carbon emissions trading and green financial instruments synergistic? -Comprehensive quantitative research based on content analysis
Source: PLoS One. 2024 Mar 7;19(3):e0298601. doi: 10.1371/journal.pone.0298601 (PMC10919601; doi:10.1371/journal.pone.0298601)
Supplement: S1 Data — (ZIP) [file pone.0298601.s001.zip › Data/地级市Z_F工作报告.zip!/╜¡╦╒/╨∞╓▌╩╨/└┤╫╘╣1⁄2╓┌║┼í╛┬φ┐╦╩2╛▌═°í┐_2015.docx]

各位代表：

现在，我代表市人民政府向大会作工作报告，请予审议，并请市政协各位委员和其他列席人员提出意见。

一、2014年工作回顾

过去的一年，面对复杂多变的宏观环境、经济下行的外部压力和各种风险挑战的叠加冲击，我们深入贯彻落实党的十八大、十八届三中四中全会和习近平总书记系列讲话精神，牢牢把握稳中求进的总要求，推动改革创新，加快转型升级，办好民生实事，效率徐州、活力徐州、幸福徐州、文化徐州和美丽徐州建设取得新进展，基本完成十五届人大三次会议确定的目标任务。

一年来，我们强化改革创新，发展内生动力明显增强

政府职能转变和行政体制改革加速推进。大力实施简政放权，取消和下放市级行政审批项目73项；出台市级行政审批事项目录清单及管理办法、加强事中事后监管意见，审批事项精简30%；编制完成行政权力清单、行政事业性收费项目目录和政府性基金目录。实施卫生计生部门整合、食品药品监管部门重组和工商质监部门管理体制调整。第一批492家市直事业单位分类工作全面完成。市级机关社会化服务综合改革持续推进。主城区街道办事处管理体制改革及扩权强镇改革、经济发达镇行政管理体制改革试点稳步推进。政府收支和政府性债务全部纳入预算管理，政府性债务规模适度、结构趋优、成本趋降、风险可控。

经济社会领域改革有序实施。实施注册资本登记制度改革，正式启动“三证合一”登记改革，新登记各类企业15602户，注册资本总额624.2亿元，同比分别增长39.2%和130%。积极推进国有企业分类改革。“营改增”试点范围进一步扩大。政府购买公共服务试点拓展至20个领域，在全省率先推行政府购买第三方绩效评价服务。2个县（区）、5个镇农村土地承包经营权确权登记颁证试点工作进展顺利。稳妥推行“单独二孩”政策。15家县级医院全面实施价格综合改革。27所学校开展名校托管试点。

重点领域创新进一步深化。放大政府资源、资产、资本、资金乘数效应，打出系列融资创新“组合拳”；率先探索政府和社会资本合作机制，2项工程入选财政部首批30个PPP模式示范项目；发行全国首单保障房资产证券化产品，设立总规模近百亿元的股权基金和产业基金；非信贷融资工具融资108亿元，市平台公司融资总规模同比增长72.3%。大力推动企业上市，新增主板、创业板首发上市企业3家，总数达10家；新增场外市场挂牌企业53家，总数翻一番。全力破解用地瓶颈，盘活存量土地8276亩，黄河故道流域土地综合整治、工矿废弃地复垦利用试点等工作成效明显，节约集约用地机制初步建立。积极探索不动产登记工作，徐州被列为全国不动产统一登记基础技术研究四个试点城市之一。全面启动“智慧徐州”建设，时空信息云平台方案通过国家测绘局专家评审，获批国家信息消费试点城市。社会信用体系加快构建。引进高层次创新创业团队13个、双创人才325人，新增国家级科技企业孵化器2家、国家高新技术企业67家、省级以上研发机构31家、江苏名牌产品22个，科技研发投入占地区生产总值比重提升到1.9%。

一年来，我们力推转型升级，经济发展稳中有进

主要指标实现平稳增长。全市完成(预计，下同)地区生产总值4900亿元、增长10.5%以上；公共财政预算收入472.3亿元、增长11.7%，税收收入占比81.8%、提高1.1个百分点；固定资产投资3672亿元、增长18.8%，其中工业投资1977亿元、占总投资的53.8%；社会消费品零售总额1664亿元、增长13%。进出口总额61亿美元，其中出口48亿美元。经济运行保持在合理区间，主要指标增幅连续九年高于全国全省平均水平。

结构调整出现积极变化。服务业完成增加值2150亿元、增长11.5%，占比达到43.5%、提高1个百分点；服务业投资增速高于总投资3.5个百分点。规模以上工业实现增加值2514亿元、增长11.2%；高新技术产业、新兴产业产值占规模以上工业比重分别达到34.7%和37.1%。粮食生产实现“十一连丰”；新增设施农业面积14.7万亩，总量、占比均居全省第一，新建4个千亩以上丘陵高亢地区设施农业示范基地；黄河故道综合开发取得阶段性成效，全长197公里的中泓疏浚贯通，“一带九区十四园”建设初具规模。民营经济发展态势较好，增加值增速高于地区生产总值增速0.8个百分点。徐州经济技术开发区获批国家级生态工业示范园区。

项目建设力度不断加大。年度实施的180项重大项目完成投资920亿元，铁路履带底盘、徐工建机塔机技改、中宇光伏黑硅电池片、考伯斯煤焦油一体化等49个项目竣工投产，徐工重型汽车、中能技改、必康新医药、雨润物流中心等重大项目有序推进，中电投风力发电站等前期项目提前开工建设。先后举办十七届投资洽谈会、徐交会以及香港、台湾、日本、新加坡等10余项重大招商活动，实际到帐注册外资17亿美元、增长13%左右，超额完成年度目标。

一年来，我们统筹城乡建设，新型城镇化扎实推进

中心城市功能日益增强。完成城市总体规划修改报批和交通发展战略、市区快速路系统、绿地系统、公共服务设施布局等专项规划编制。8大类258项城建重点工程开工232项、竣工116项，开工率90%。三环东路高架快速路建成通车，城市轨道交通1号线、三环西路和三环北路高架快速路、观音机场二期、徐明高速开工建设，郑徐客运专线建设、徐宿淮盐铁路前期工作进展顺利，徐州综合交通枢纽地位进一步凸显。中心商圈、高铁生态商务区二期、新城区“四轴一片区”等重点片区开发，以及八里家居博览中心、淮海文博园二期、铜山万达广场、徐州保税物流中心等项目扎实推进。刘湾水厂改扩建工程建成通水，骆马湖水源地及第二水厂动工兴建。实施棚户区改造项目52个，总面积1005万平方米、是前三年改造总量的1.5倍。

城市管理水平全面提高。全力开展国家卫生城市和生态园林城市创建活动，老旧小区、背街小巷、“六小行业”等10项专项治理成效显著，城市长效化网格化管理机制进一步完善，顺利进入国家卫生城市公示名单。周密部署非法营运电（机）动三（四）轮车集中整治，城区交通秩序明显改观。城区生活垃圾日产日清、无害化处理率达到100%。“外创示范道路，内创幸福家园”活动深入开展，新增5条省级示范路、2个省级示范社区，幸福家园创建获得省人居环境范例奖。

新型城镇体系加快形成。五县（市）累计完成征迁面积900万平方米，开工建设安置房700万平方米。30个重点中心镇和特色小城镇规划全面完成，命名第三批7个创建达标中心镇和首批13个管理示范镇。完成村庄环境综合整治4103个，创建三星级“康居乡村”59个、新农村示范村51个。建成农村公路493公里、桥梁105座。改造农村危房6300户。完成农田水利投资10.5亿元。实施农村饮水安全工程，新增受惠群众59.9万人。

一年来，我们坚持绿色发展，生态建设迈出坚实步伐

大气和水污染防治持续强化。完成77项大气污染防治年度重点工程，淘汰4.1万辆老旧机动车，秸秆禁烧与综合利用取得明显成效，全市环境空气质量二级以上天数238天、比去年增加46天，PM2.5平均浓度下降12.6%。投资13.5亿元实施水环境治理，推行城镇河道保洁养护市场化。京杭运河沿线190家非法码头整治全部到位。全市污水集中处理率89%，功能区三类以上水体占比76.6%。南水北调国控断面实现稳定达标。

绿色徐州建设成效明显。“二次进军荒山”计划全面完成，新增造林15.8万亩，林木覆盖率达到32%、高于全省平均水平10个百分点；市区新增绿地110公顷，300亩以上大型开放式园林达到27个，人均公园绿地超过17平方米，建成区绿化覆盖率提高到43%。云龙湖风景区通过国家5A级景区资源景观质量评审，新增国家4A级景区8家、省级生态旅游示范区3家。徐州城市形象明显改观，呈现出“一城青山半城湖”的新景象。

节能减排和生态市创建进展良好。依法关停小钢铁企业57家，整合替代城区热电企业3家，主城区化工企业全部实现退城入园，年耗能3000吨标准煤以上企业的节能改造全面完成。单位GDP能耗下降4%以上，完成节能减排年度任务。我市入围国家节能减排财政政策综合示范城市。沛县通过国家生态县技术评估，铜山、贾汪两个区通过省级生态县（区）命名，43个镇成为国家级生态镇。

一年来，我们突出民生为先，和谐社会建设取得新的成效

100项民生实事工程基本完成。新建社区居家养老服务站点235个、城市小型托老所12个、农村老年关爱之家54个、妇女儿童之家100个；各类养老机构达250家，总床位数4.2万张。为主城区10万名60周岁以上老人集体购买意外伤害综合保险。救助特殊困难残疾群众41万人次。建设残疾人托养机构146个，市级残疾人康复中心竣工。徐州殡仪馆有序搬迁，殡葬整治稳妥实施。更新公交车辆200辆，新增出租汽车465辆，城区公共交通出行分担率达26%。农贸市场改造、便民早餐工程等项目顺利推进。

居民收入和就业稳定增长。城乡居民收入分别提高到26100元和13350元，增长10%和11.5%。新增城镇就业11.7万人，失业人员再就业8万人，农村劳动力转移6.4万人，城镇登记失业率1.91%；城乡劳动者职业技能培训7.8万人、创业培训3.5万人；建成大学生创业园19个、培育创业企业5100余家。扶贫开发取得阶段性成果，27万低收入人口实现脱贫。

社会保障水平持续提高。全市社会保险参保人数650万人，社保卡持卡人数310万人，社保基金收支总额363亿元，主要险种参保覆盖率均超过95%。新农合参合率100%，筹资标准和财政补助标准进一步提高。城镇居民、职工医保政策范围内住院报销比例分别达到72%和83%。市级大病保险制度和大重病患者长效补助机制基本建立。城乡低保标准分别提高到每人每月558元和320元，城市“三无”老人保障标准提高到每人每月1223元。开工建设保障性安居房6926套、建成7914套，超额完成省定任务。

各项社会事业协调发展。教育“三区同建”加快推进，以市为单位在苏北率先全面通过“全国义务教育发展基本均衡县(市、区)”国家级验收；新建改建中小学40所、幼儿园102所，加固重建中小学校舍72万平方米。完成33个社区卫生服务中心提档升级，创建国家级示范社区卫生服务中心3个、省级示范社区卫生服务中心21个、省级示范乡镇卫生院51所，全市三级医院增至16家。举办首届徐州文化博览会，广播电视户户通等文化惠民工程扎实开展。成功承办第十八届省运会，夺得赛会金牌、奖牌、总分三项第一；晋身省体育强市和全国武术之乡。出生人口性别比进一步降低，人口自然增长率控制在6‰以内。第三次经济普查全面完成。人防、双拥、国防动员、民族宗教、档案、外事、侨务、台湾事务、审计、物价、新闻出版、应急管理、住房公积金管理、无线电管理、淮塔管理、防震减灾、气象、青少年、妇女儿童、关心下一代等各项事业都取得了新进步。

一年来，我们践行群众路线，政务环境持续优化提升

机关作风取得明显改善。扎实推进政府系统党的群众路线教育实践活动，认真开展办公用房清理、“吃空饷”清理等十项重点专项整治，压缩会议1021个、文件2042个、评比达标表彰274个，撤销各类领导小组和协调机构1154个、核减因公临时出国出境111批次428人。制定政府系统建章立制项目22个，“两个习惯”作风建设深入开展，行风政风建设进一步强化。市本级“三公”经费下降20.3%，市直101家单位预决算全部公开。

政府效率效能加速提升。继续深化百姓办事“零障碍”工程，全面推行“两参一改”监督评议，共清理办事障碍3220项、优化办事流程165项、完善办事制度412项。加快推进电子政务建设，政务协同办公系统覆盖235个部门单位，政府公文实现网络交换、及时办理，流转时间大幅缩短，初步建立网络化、无纸化政务办公新机制。拓展完善“12345”政府服务热线功能，接处来电43万件，办结率97.4%。法治政府建设不断强化，建立政府参事制度，政务决策程序更加规范。自觉接受市人大及其常委会的法律监督和工作监督、市政协及社会各界的民主监督，办理人大代表建议287件、政协提案494件，答复率100%、满意率98%以上。

社会治理能力全面加强。健全城乡社区三级服务平台，新建改建县（市）区服务中心173个，全市城乡和谐社区达标率分别提升到85%、75%，政社互动试点、社区减负增效和“三社联动”工作有序开展。深入推进平安建设、法治建设，扎实开展恶意骗保企业资产“百日追讨”活动和非法集资处置工作，涉法涉诉信访改革和行政执法、刑事司法“两法衔接”走在全省前列，徐州特色大调解模式初步构建，刑事案件发案率下降22.4%，跻身全国“六五”普法中期先进城市。

各位代表，政府工作的每一份成绩，都离不开全市上下的共同努力，离不开社会各界的大力支持。在此，我代表市人民政府，向全市各族人民群众，向人民解放军驻徐部队、武警官兵和公安民警，向各民主党派、工商联、无党派人士和人民团体，向支持徐州现代化建设的香港特别行政区同胞、澳门特别行政区同胞、台湾同胞、海外侨胞和各界人士，向投身徐州经济社会发展的国内外投资者和企业家，表示衷心的感谢！

在总结成绩的同时，我们也必须正视徐州当前发展面临的突出问题：经济增长动力不足、下行压力较大，进出口总额、城镇居民收入等指标增速与年初预期存在一定差距；转型升级任务依然艰巨，结构调整阵痛初显，一些传统优势产业出现产能过剩，部分企业生产经营困难；城镇化水平有待进一步提升，城乡二元结构依然明显，镇村基础设施和公共服务体系建设相对滞后；生态环境仍较脆弱、承载能力接近饱和，城市管理还存在一些薄弱环节；财政收支平衡压力加大，社会保险基金征缴扩面还需进一步强化，民生领域的部分遗留问题尚未妥善解决，影响社会和谐稳定的矛盾依然存在；有的领域改革攻坚力度还不够大，部分改革举措落地见效还不够快；政务服务效能还有较大提升空间等等。对此，我们必须高度重视，认真加以解决。

二、2015年目标任务和工作重点

今年是全面完成“十二五”的收官之年、全面深化改革的关键之年、全面推进依法治国的开局之年。从国际环境看，世界经济仍处在金融危机后的深度调整期，全球增长格局、国际分工趋势、投资贸易规则、地缘政治环境等都在发生深刻变化，总体复苏疲弱态势难有明显改观。从国内形势看，我国经济发展进入新常态，国家为适应新常态出台了一系列重大政策，特别是加强地方政府性债务管理、清理规范税收等优惠政策、创新重点领域投融资机制鼓励社会投资等举措实施后，对地方政府投融资、城市建设、招商引资、经济运行都将产生重大影响，靠传统思维方式和惯用方法手段推动发展已难以与新常态相适应。

对于徐州来讲，当前既拥有综合交通枢纽、区域性中心城市功能不断提升的优势，又面临着国家推进“一带一路”建设的重大机遇，更面临着新常态下诸多新的挑战：需求结构上，我市出口总量较小，投资带动力有所减弱，消费潜力有待进一步挖掘；电子商务对传统消费空间形成挤压，对徐州区域性商业中心的地位带来一定冲击。产业发展上，传统优势产业规模已邻近“天花板”，受市场空间制约的效应已经开始显现，而新兴产业整体实力不强、竞争力不够，科技创新能力不足，新的增长支撑点尚未形成。要素保障上，依靠大量拼资源、消耗土地和牺牲生态环境的发展路子已无法持续，而徐州是老工业基地和资源枯竭型城市，产业结构仍然偏重，资源、环境对发展的约束更加明显。

新常态需要新思路，新发展需要新举措。我们要认真学习贯彻习近平总书记视察江苏重要讲话精神，深刻认识、主动适应、勇于引领新常态，围绕建设“经济强、百姓富、环境美、社会文明程度高”的新徐州，顺应国家政策导向，趋利避害、顺势而为，主动调整发展思路、创新工作方式，在新常态下实现更高质量、更可持续、更加健康的发展。要以国际化视野引领开放，主动对接上海自贸区，融入“一带一路”建设，放宽外资进入领域，推动境外企业和本地产业对接融合，更高水平“引进来”、更大步伐“走出去”，加快培育一批国际化企业，拉长开放型经济“短板”。要以市场化方式调控运行，深化行政审批制度改革，加快政府职能转变，最大限度减少政府对微观事务的管理，更加善于运用市场手段破解难题、推进项目建设、加快结构调整、促进百姓创业，真正让市场在资源配置中起决定性作用。要以城镇化进程统筹发展，一方面继续提升城市功能，更加注重发展城市产业，推进产城融合，切实增强城市的凝聚力和吸纳力；另一方面以完善镇、村布局为突破口，切实加大农村投入，推动现代农业再上新台阶，形成以城带乡、城乡互动、一体发展的新格局。要以信息化手段推动转型，以“智慧徐州”建设为抓手，深入实施创新驱动战略，将信息技术广泛运用于产业改造升级、城市建设管理和社会事业发展，推进信息化和新型工业化、城镇化深度融合，提高经济运行效率、产业综合素质、城市管理水平和群众生活质量。要以低碳化要求倒逼升级，以能源消耗总量控制为倒逼机制，加大落后产能淘汰、污染治理和环境保护力度，大力发展低碳产业，推动发展模式由“高投入、高排放”向“高效益、低排放”转变，创造更加舒适宜居的环境。要以法治化思维治理社会，进一步强化法治观念、践行法治精神，实现权力授予法定化、权力运行规范化、权力监督常态化，依法维护人民权益、维护公平正义、维护社会安全稳定，全面推进依法治市、努力建设法治社会。

今年经济社会发展的主要预期目标是：（1）地区生产总值增长10%左右；（2）一般公共预算收入增长10%左右；（3）固定资产投资增长17%左右；（4）社会消费品零售总额增长13%左右；（5）实际到帐注册外资、进出口总额保持增长；（6）全体居民人均可支配收入增长10%左右；（7）居民消费价格涨幅不高于全省平均水平；（8）科技研发投入占地区生产总值比重达2%左右；（9）城镇登记失业率控制在4%以内；（10）节能减排完成省定任务；（11）人口自然增长率控制在6‰以内。

我们将重点抓好六个方面的工作：

（一）千方百计增进民生福祉，顺应人民群众生活新期待。坚持尽力而为、量力而行，深入实施民生幸福工程，办好11大类90件民生实事，不断提升群众幸福感和满意度。

继续完善社会就业网。强化人力资源市场建设，启用就业统计分析决策系统，开展农村劳动力转移景气指数试点。统筹做好大学毕业生、城镇就业困难人员、退役军人等重点人群就业工作，实现就业精准服务，确保新增城镇就业8万人、城镇失业人员再就业4万人、农村劳动力转移5万人。坚持“输血”和“造血”相结合，创新扶贫开发机制，推动全市年人均收入低于4000元的农村居民实现全部脱贫。

不断健全社会保障网。启动机关事业单位养老保险改革，推行“全民参保登记计划”，做好参保扩面和社保基金清欠征缴，强化被征地农民社会保障，完善覆盖城乡居民的社保体系。改进企业养老保险缴费基数申报方式，严格实施社会保险费“五险一票”征收。提高新农合人均筹资标准和保障水平。为城镇居民医保参保人员投保大病保险。进一步提高城市“三无”老人、孤儿、农村五保对象供养及城乡低保以标准，为全市五保对象购买重大疾病商业保险，继续为主城区60周岁以上老人集体购买意外伤害综合保险。加快住房保障体系建设，新开工公共租赁住房2180套，基本建成2510套。

努力织密公共服务网。实施全面改善义务教育薄弱学校基本办学条件工程，新建改扩建幼儿园80所、中小学校35所，加固中小学校舍53万平方米，创建省义务教育现代化学校100所；健全中职、高职、本科有机衔接的培养机制，确保通过省“职业教育创新发展实验区”验收。抓好北区股份制医院等4家市区综合性医院建设，实施新一轮城市社区卫生服务机构提档升级工程，加强城乡基层医疗卫生机构规划建设；推进分级诊疗，规范医疗服务。完善公共文化服务体系，继续办好徐州文化博览会，打响“书画徐州”等文化品牌，推动文化产业加快发展。安装100处公共场所体育健身设施，加快建设体育公共服务体系示范区和国际武术文化名城，促进全民健身和竞技体育协调发展。继续稳定低生育水平，加强流动人口计划生育管理服务，综合治理出生人口性别比偏高问题。推进军民融合式发展，确保全国双拥模范城创建“八连冠”。重视发展老龄事业，继续抓好养老服务中心建设，新建20个农村老年关爱之家；推进公办养老机构“公建民营”改革，发展“医养结合”的养老服务机构。加强殡葬管理服务，落实殡葬惠民政策，加快平价公墓和农村公益性公墓建设。统筹抓好其他各项社会事业。

坚决筑牢社会安全网。加强基层基础工作，推进城乡社区服务管理网格化，新建改扩建社区（村）综合服务中心113个，力争城乡社区网格化服务管理达到80%以上。继续开展社会矛盾大排查大调解，全面推行“阳光信访”和信访法治化，完善“多调对接”人民调解徐州模式，推动矛盾及时就地化解。抓好社会治安技防监控工程，依法打击各类违法犯罪活动。深入开展“质量促进年”活动，加快产品质量公共服务平台建设，强化对事关群众健康和安全的产品质量监管，建设质量强市。完善食品药品生产、流通、消费全环节监管体系，让市民吃上放心食品。坚持“党政同责、一岗双责、齐抓共管”，严格落实安全生产责任，强化应急管理，加强公共安全风险评估和防控，有效防范、坚决遏制各类安全事故发生。

（二）坚定不移推进改革攻坚，释放经济社会发展新活力。围绕解决发展面临的突出问题，深化重点领域的改革，最大限度激发各方面的积极性和创造力。

以政府职能转变为中心加快推进行政体制改革。继续深化行政审批制度改革，在完成“五张清单”编制工作的基础上，建立清单动态监管和事中事后监管机制，构建与权力清单相配套的权界清晰、分工合理的部门职责体系。继续简化审批手续、优化权力运行流程，推进审批事项和政府公共服务项目全部进驻政务服务中心，完善公共资源交易中心和政务服务中心一体化管理机制。巩固市、县政府机构改革成果，继续做好省级经济发达镇行政管理体制改革、市级扩权强镇改革试点和街道办事处管理体制改革。适时启动行政类和生产经营类事业单位改革，开展事业单位法人治理结构试点。严格控制机构、编制、财政供养人员总量和领导职数。完成公车改革任务。积极培育、规范发展各类社会中介组织，实现行业协会商会类社会组织同行政机关全面脱钩。积极推进区域交通一体化改革试点。

以发挥市场作用为重点进一步深化经济体制改革。深入推进国有企业分类改革，制定出台国有企业领导人员管理及市场化选聘方案，稳妥实施市属企业重组整合方案，推进经营性国有资产集中统一监管。支持大型国有企业创新投融资模式，引进战略投资者，优化股权结构，完善激励约束机制。创新重点领域投融资机制，在公共服务、资源环境、生态保护、基础设施等领域，推广政府和社会资本合作模式，增强公共产品供给能力。优化政府投资使用方向，改进政府投资使用方式，通过投资补助、基金注资、担保补贴、贷款贴息等方式，支持社会资本参与重点领域建设。加快推进黄河故道流域土地综合整治试点，加大工矿废弃地复垦利用力度，规范实施城乡用地增减挂钩。切实抓好不动产统一登记基础技术研究试点。

以建立现代财政制度为方向推进财税体制改革。改进预算管理制度，将一般债务收支纳入一般公共预算管理，将专项债务收支纳入政府性基金预算管理，将政府与社会资本合作项目中的财政补贴等支出按性质纳入相应政府预算管理，建立跨年度预算平衡机制。继续扩大“营改增”试点范围。清理规范税收等优惠政策，切实加强税收征管。严格财政支出管理，强化支出预算约束，全面推进预算绩效管理，进一步提高财政资金使用效益，拓展政府购买公共服务领域。建立规范的地方政府举债融资机制，剥离融资平台公司政府融资职能，融资平台公司不再新增政府债务。

以优化基本公共服务为目标抓好社会领域改革。加大教育、医疗、文化等社会领域对外合作开放力度，创新合作方式，鼓励引导外资、民资参与社会事业发展。推动幼教集团股份制改造试点，引进第三方评价机构开展办学水平和教育质量监测评估。深化医疗保障、医疗服务、公共卫生、药品供应、监管体制综合改革，积极推进市县两级公立医院改革和新沂综合医改创新试点，加快建立现代医疗卫生体系。深化国有文化单位改革，鼓励文化企业和社会力量共同参与文化建设。探索通过社会化收储、发放货币补贴或直接收购等方式筹集保障性住房，完善保障房退出和公租房运营管理机制。加快社会信用体系“三库一网一平台”建设。

（三）全力以赴转方式调结构，增创徐州科学发展新优势。在努力保持稳定增长的同时，加快经济结构战略性调整，积极培育新的增长点，打造徐州经济“升级版”。

加大重大项目建设和引进力度。继续发挥“三重一大”的龙头带动作用，进一步扩大投资规模、优化投资结构、撬动社会资本投入。年内计划安排重大项目180项、总投资3592亿元，其中当年实施156项、年度投资782亿元。扎实推进郑徐客运专线、徐宿淮盐铁路、观音机场二期、骆马湖水源地及第二水厂等23项基础设施项目，加快实施徐工重型汽车、升华电梯制造、恒盛化工技改、苏宁商务广场、润成新能源汽车等133项重大产业项目，努力把淮海新能源汽车、三星机械精密机床等24项前期推进项目转化为新开工项目，为保增长调结构提供有力支撑。在支持本地企业增资扩股的同时，适应新常态下招商工作形势，转变招商理念、创新招商方式，继续加强产业链招商、专题招商和以商招商，进一步提升招商团队在出国（境）人员中的比重，提高重点招商活动实效。

鼓励支持全民创业。以建设创业型城市为契机，完善创业扶持政策，引导高校毕业生、返乡农民工等重点群体开展自主创业活动。发挥徐州高等教育资源和职业教育资源相对集中的优势，联合行业企业和中介组织，建立社会化创业培训体系，提高创业培训实效，完成城乡劳动者职业技能培训4万人、创业培训6000人。深入实施“彭城英才计划”和“创新型企业家培育计划”，大力引进培养高层次创新创业人才、高水平管理人才、高技能实用人才和创新创业团队。依托开发区、高新区、工业园区和大学科技园，因地制宜打造创业实训基地和孵化平台。强化创业指导，为创业者提供政策咨询、信息发布、项目推介、风险评估、贷款融资、注册登记等服务，努力降低创业风险，提高创业成功率。

增强企业核心竞争力。以做大做强做优骨干企业为目标，鼓励支持优势企业通过兼并、收购、联合、参股等多种形式开展投资合作，支持有条件的企业开拓外部市场、实施境外并购，培育具有国际竞争力的企业群体。全力扶持一批高成长型、科技型、创业型的中小企业，支持中小企业与优势企业开展协作配套，促进其向“专精特新”方向发展，培育一批“行业小巨人”。全面推进个体工商户转企业法人和小规模纳税人转一般纳税人工作，助推各类小微企业转型升级。继续落实中小微企业金融顾问制度，完善企业信用担保体系，努力解决中小企业融资难问题。加强企业股份制改造和上市后备企业梯队建设，引导企业完善治理结构、创新商业模式、抓好品牌创建、提升技术创新能力，打造“百年企业”。

加快主导产业提档升级。坚持不懈做好“加、减、乘”法，促进产业链、价值链和创新链同步提升，形成若干有竞争力的产业链和产业集群。做好加法，重点是改造提升传统优势产业。着力增强装备制造业自主研发、制造和成套能力，提高食品产业加工增值、安全保障、品牌创建水平，推动能源产业向煤电、煤电建材、煤电化工等一体化发展。做好减法，重点是完善落后产能退出机制。按照消化一批、转移一批、整合一批、淘汰一批的思路，分类抑制化解钢铁、水泥等行业严重过剩产能，加快推进煤盐化工、冶金、建材产业优胜劣汰、整合重组，腾出环境容量用于支持高端产业发展。做好乘法，重点是加快发展新兴产业和现代服务业。继续实施培育和发展新兴产业三年行动计划，多渠道加大科技创新投入，主攻关键技术创新和产品推广应用。抓好徐州商圈规划建设，优化调整服务业集聚区布局，推动传统服务业扩容提质，促进旅游业等现代服务业加快发展，积极培育新的消费热点，力争服务业比重再提高1个百分点。把握互联网经济发展新趋势，抓好农村电子商务“镇村通”改革试点，推广“沙集模式”，推动电子商务和物流、邮政快递等业态联动发展，高水平建设全国电子商务示范城市。

提升集约集聚发展水平。强化开发园区经济发展主阵地作用，加大简政放权力度，促进各个园区提升承载能力，实现错位发展。推动徐州经济技术开发区加快高铁生态商务区二期、工程机械和新能源汽车产业园等功能园区建设，力争保税物流中心通过验收，启动国家综合保税区创建工作；支持徐州高新区和各省级开发区因地制宜打造特色产业园，建设公共创新服务平台、科技研发中心和创业孵化器，积极申创创新园区及循环经济园区，提高开发园区对全市经济的贡献率。建立健全国土资源节约集约利用综合评价考核制度，开展批而未用土地专项清理，加大闲置用地处置力度，大力推进高标准厂房建设，加快工矿废弃地等存量土地盘活利用，提高工业用地投资强度和产出强度；做好城市地质调查，为地下空间规划和开发利用打好基础。

（四）扎实推进新型城镇化，构筑城乡一体发展新格局。高水平规划建设现代城市、完善城镇体系，打造区域性“八大中心”，促进城乡共同繁荣。

大力提升中心城市功能。完善各类专项规划和控制性规划，探索“多规合一”，实施150项城建重点工程，增强城市功能和辐射带动力。围绕构建立体化交通体系，抓好轨道交通一期工程、三环西路和三环北路高架快速路建设以及城区15条道路改造，加快黄河故道开发公路等干线路网建设，促进路网互联互通。立足城市功能配套化，抓好高铁客运站、北区长途客运站、公交首末站、7个农贸市场改造、4类城市水务、7类环卫设施等项目建设。大力推进棚户区及城中村改造，实施老旧小区整治“回头看”，进一步改善城市面貌。突出城市建设文化内涵，加快户部山历史文化街区建设，保护好钟鼓楼等历史文化古迹，彰显徐州“楚韵汉风、南秀北雄”的城市特质。

促进区域经济联动协调发展。突出工业兴县，扎实推进新一轮“丰县崛起”行动计划，深入实施“突破睢宁”、沛北一体化战略，加快新沂、邳州新型工业化进程，完善县城区医疗、教育、就业、环境配套功能，切实强化中心城区与各县城区在交通、产业、公共服务上的对接。鼓励城区错位发展，支持鼓楼区老工业区搬迁改造试点、云龙区省级服务业综合改革试点，积极推进泉山区33平方公里产城生态一体淮海新城建设，加快贾汪区资源枯竭城市转型发展、铜山区与主城区全面融合，完善新城区配套设施，促进城市综合开发与发展现代服务业互动并进，加快产城融合进程。

全面提高乡镇建设水平。继续推动30个中心镇建设，再创10个左右管理示范镇。加快完善城镇水、电、路、气、通讯等基础设施，配套建设学校、医院、市场、公交等公共服务设施，增强中心镇承载能力。依托资源优势和现有基础，按照“一镇一业”的要求，打造一批工业强镇、商贸大镇、旅游名镇。适度修编一般镇总体规划，优化农村居民点空间布局，完善农村基础配套，切实提高城乡一体化水平。

（五）加快农业现代化步伐，推动农业农村工作迈上新台阶。在稳定粮食生产的同时，大力提高农业综合效益、组织化程度、科技进步贡献率、农产品竞争力，让徐州的农业更强、农民更富、农村更美。

加大农村改革创新力度。全面开展农村土地承包经营权确权登记颁证，建立健全农村土地流转市场，完善土地流转机制。吸引工商资本参与现代农业发展，扩大适度规模经营。加快农村产权制度改革，推进农村产权交易市场试点，规范完善农村社区股份合作社内部管理制度，加快形成农村资产资本化、农村资源市场化、农民增收多元化新格局。深入推进小型水利工程管理体制改革。积极推进农村土地制度综合改革，深化农村集体经营性建设用地、农村宅基地、征地制度改革。

积极构建现代农业经营体系。深入实施“2020”工程，新建改造20万亩设施农业、20个规模养殖场。突出抓好黄河故道沿线农业综合开发和丘陵高亢地区设施农业示范工程，加快7个省级以上农产品加工集中区建设，扶持壮大一批农产品加工龙头企业，提升农产品附加值。加快培育专业种养大户、家庭农场、农民合作社等新型农业经营主体，开展新型职业农民教育培训，健全农业社会化服务体系。强化市县农产品智能监管平台建设，保障农产品质量安全。

着力改善农业农村基础设施。开展粮食高产示范创建，实施灌区节水改造、农村河道疏浚整治、山区水源工程、规模化高效节水工程等10项农村水利工程，新增有效灌溉面积10万亩、旱涝保收农田面积13万亩、节水灌溉面积35万亩。大力发展农业装备，力争农业综合机械化水平达到80%。新建新农村示范村50个，建设农村公路200公里、桥梁100座，提升镇村公交开通率，解决70.1万名农村居民饮水安全问题，抓好1600多个村庄的环境整治工作，让农村成为干净整洁的美丽家园。

（六）坚持不懈抓好生态文明建设，彰显美丽徐州新形象。以“五大行动计划”为抓手，强化污染防治和生态修复，全力争创国家生态园林城市。

加强节能减排和环境保护。抢抓被列入国家节能减排财政政策综合示范城市的机遇，科学编排、认真落实节能减排项目，确保完成省下达的年度任务和“十二五”节能减排目标。继续抓好工业企业退城入园，强化燃煤污染、工业污染、扬尘污染、机动车污染控制，抓好秸秆禁烧与综合利用，加大搅拌站、散货堆场整治，加快热电整合步伐，确保完成PM2.5平均浓度下降考核目标。全面完成34项国家治污工程，继续抓好省确定的281项治污工程建设，确保重点断面水质稳定达标。加快推进生态市创建，积极争取国土资源生态文明建设试点，力争各镇全部通过国家生态镇考核验收。

打造整洁有序的城市环境。巩固提升卫生城市创建成果，突出智慧城市管理，健全占道经营、违章建筑、“六小行业”等长效治理机制，完善公厕、垃圾收运等基础设施，坚决防止脏乱差现象“回潮”。深入开展电（机）动三（四）轮车非法营运治理，优化城市交通秩序，缓解交通拥堵问题。实施市容环境专项整治工程，加强对公共场所、背街小巷以及城乡结合部的整治。深入开展“外创示范道路，内创幸福家园”活动，重点提升道路规范化管理和小区物业管理水平。引导居民摒弃陋习、提升文明素养，加快创建全国文明城市。

积极挖掘生态旅游效益。发挥生态建设品牌效应，注重把生态资源转化为旅游资源，培育壮大旅游产业，实现生态效益和经济效益同步提升。充分利用独特的山水生态资源和汉文化特色，以云龙湖5A级景区创建为龙头，整合汉文化资源，规划一批精品线路，打造徐州旅游品牌，努力形成淮海经济区旅游目的地城市。加快微山湖、潘安湖、吕梁山等景区建设步伐，完善旅游配套设施，培育一批生态游、乡村游热点。推进旅游景区公司化运营改革，加快建设徐州（省级）游客集散中心和智慧旅游平台，构建市、县（市）区分级承运体系，着力提升旅游公共服务水平。

三、加强和改进政府工作

全面完成今年各项目标任务，对政府自身建设提出了更高要求。我们必须加快转变政府职能，创新行政管理方式，进一步提升政府工作的创造力、执行力和公信力。

严格依法行政，提高法治政府建设水平。坚持“法定职责必须为、法无授权不可为”，依法全面履行政府职能。健全依法决策机制，把公众参与、专家论证、风险评估、合法性审查、集体讨论决定作为重大行政决策的法定程序，加强重大决策跟踪反馈和责任追究，确保行政决策权限合法、实体合法、程序合法。深化行政执法体制改革，完善行政裁量权基准制度，落实行政执法责任制，推进综合执法，提高执法效率和执法能力。认真执行市人大及其常委会的决议和决定，自觉接受人大工作监督、法律监督和人民政协民主监督，主动听取各民主党派、工商联、无党派人士、人民团体、政府参事的意见建议，加强审计监督，高度重视人民群众和新闻舆论监督。

深化政务公开，提高阳光政府建设水平。围绕决策、执行、管理、服务、结果公开，加大重点领域和关键环节政府信息公开力度，突出抓好财政预算、公共资源配置、重大建设项目批准和实施等政府信息公开。强化信息发布，完善例行发布、应急发布、政策解读等机制，做好社会热点回应，加强互动交流。抓好互联网政务信息服务平台和便民服务平台建设，提升政府网站传播能力，推进政务公开信息化。

增强效率效能，提高服务型政府建设水平。继续实施百姓办事“零障碍”工程，拓展“12345”政府服务热线功能，完善网上政务服务，依托市行政审批服务平台，整合各职能部门网上审批资源，推行信息共享，建设全市统一集行政审批、便民服务、政务公开等功能于一体的网上政务大厅及审批服务平台，打造市县镇三级联动网上政务服务体系。坚持把狠抓落实作为政府工作的核心职责，完善绩效考评体系，强化督促检查，弘扬“钉钉子”精神，对重要事项、重大问题、重点工作抓紧盯牢。高水平编制“十三五”规划，做好统计工作，为创新发展提供支撑。

切实转变作风，提高廉洁政府建设水平。严格执行中央八项规定和省委、市委十项规定，进一步精简会议活动、文件简报，集中精力抓落实干实事谋发展。巩固和发展教育实践活动成果，牢固树立群众观点，持之以恒地反对形式主义、官僚主义、享乐主义和奢靡之风。抓好“约法三章”、“六个不准”、“九个一律”等规定的落实，强化“两参一改”和评议员常态化巡查，完善声屏网报“四位一体”行风热线媒体联动机制，进一步开展作风行风监督评议。深入开展“两个习惯”教育，让习惯在监督的环境下工作、习惯在法治的轨道上用权成为政府和行政人员的行动自觉。全面落实党风廉政建设责任制，健全岗位风险防控制度，严肃查处各类违纪违法案件，坚决纠正损害群众利益的不正之风。

各位代表！徐州的发展充满机遇，实现振兴的任务光荣而艰巨。让我们紧密团结在以习近平同志为总书记的党中央周围，高举中国特色社会主义伟大旗帜，在中共徐州市委的坚强领导下，团结依靠全市人民，敢于担当，真抓实干，为“迈上新台阶、建设新徐州”而努力奋斗！
